# 2-(4-Hy­droxy­phen­yl)-3-(trimethyl­sil­yl)propanaminium chloride

**DOI:** 10.1107/S1600536811037639

**Published:** 2011-09-30

**Authors:** Yousef M. Hijji, Ray J. Butcher, Jerry P. Jasinski, Zachary White, Robert C. Rosenberg

**Affiliations:** a Chemistry Department, Morgan State University, 1700 East Cold Spring Lane, Baltimore, MD 21251, USA; b Department of Chemistry, Howard University, 525 College Street NW, Washington, DC 20059, USA; c Department of Chemistry, Keene State College, 229 Main Street, Keene, NH 03435-2001, USA

## Abstract

In the title crystal structure, C_12_H_22_NOSi^+^·Cl^−^, anions and cations are linked *via* O—H⋯Cl, N—H⋯Cl and N—H⋯O hydrogen bonds to form a two-dimensional network parallel to (101). Within the hydrogen-bonded network, *R*_4_^2^(22) ring motifs are stacked along [010].

## Related literature

For silicon-substituted β-phenyl­ethyl amines and their biological activity, see: Frankel *et al.* (1968 ▶). For applications of β-phenyl­ethyl amine in alkaloid synthesis *via* the Pictet–Spengler reaction, see: Lorenz *et al.* (2010 ▶). For the uses and applications of 3-amino-propyl­silanes in nanotechnology and self-assembled monolayers, see: Li *et al.* (2009 ▶). For the uses and applications in reverse ionic liquids in oil extraction, see: Blasucci *et al.* (2010 ▶). For a related structure, see: Hijji *et al.* (2011 ▶). For standard bond lengths, see: Allen *et al.* (1987 ▶). For hydrogen-bond motifs, see: Bernstein *et al.* (1995 ▶).
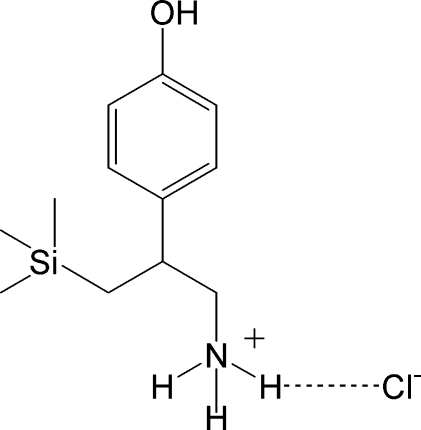


## Experimental

### 

#### Crystal data

C_12_H_22_NOSi^+^·Cl^−^
*M* *_r_* = 259.85
Monoclinic, 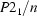

*a* = 14.2611 (4) Å
*b* = 6.7587 (2) Å
*c* = 16.0316 (9) Å
β = 91.252 (3)°
*V* = 1544.86 (11) Å^3^
*Z* = 4
Cu *K*α radiation
μ = 2.79 mm^−1^
*T* = 295 K
0.44 × 0.18 × 0.06 mm

#### Data collection

Oxford Diffraction Xcalibur Ruby Gemini diffractometer
Absorption correction: multi-scan (*CrysAlis PRO*; Oxford Diffraction, 2009 ▶) *T*_min_ = 0.713, *T*_max_ = 1.000
5741 measured reflections
3065 independent reflections
2023 reflections with *I* > 2σ(*I*)
*R*_int_ = 0.034

#### Refinement

*R*[*F* ^2^ > 2σ(*F* ^2^)] = 0.061
*wR*(*F* ^2^) = 0.244
*S* = 1.14
3065 reflections
146 parameters
H-atom parameters constrained
Δρ_max_ = 0.42 e Å^−3^
Δρ_min_ = −0.46 e Å^−3^

### 

Data collection: *CrysAlis PRO* (Oxford Diffraction, 2009 ▶); cell refinement: *CrysAlis PRO*; data reduction: *CrysAlis PRO*; program(s) used to solve structure: *SHELXS97* (Sheldrick, 2008 ▶); program(s) used to refine structure: *SHELXL97* (Sheldrick, 2008 ▶); molecular graphics: *SHELXTL* (Sheldrick, 2008 ▶); software used to prepare material for publication: *SHELXTL*.

## Supplementary Material

Crystal structure: contains datablock(s) I, global. DOI: 10.1107/S1600536811037639/lh5328sup1.cif

Structure factors: contains datablock(s) I. DOI: 10.1107/S1600536811037639/lh5328Isup2.hkl

Supplementary material file. DOI: 10.1107/S1600536811037639/lh5328Isup3.cml

Additional supplementary materials:  crystallographic information; 3D view; checkCIF report

## Figures and Tables

**Table 1:** Hydrogen-bond geometry (Å, °)

*D*—H⋯*A*	*D*—H	H⋯*A*	*D*⋯*A*	*D*—H⋯*A*
O1—H1⋯Cl^i^	0.82	2.23	3.012 (4)	160
N1—H1*A*⋯Cl^ii^	0.89	2.39	3.146 (3)	143
N1—H1*B*⋯O1^iii^	0.89	2.18	2.941 (5)	143
N1—H1*C*⋯Cl	0.89	2.21	3.093 (4)	172

Symmetry codes: (i) 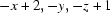; (ii) 
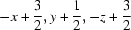
; (iii) 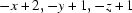.

**Fig. 1.**
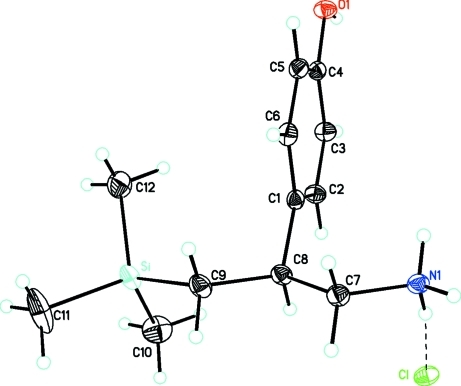
The asymmetric unit of the title compound. A hydrogen bond is shown by a dashed line (30% atomic displacement parameters).

**Fig. 2.**
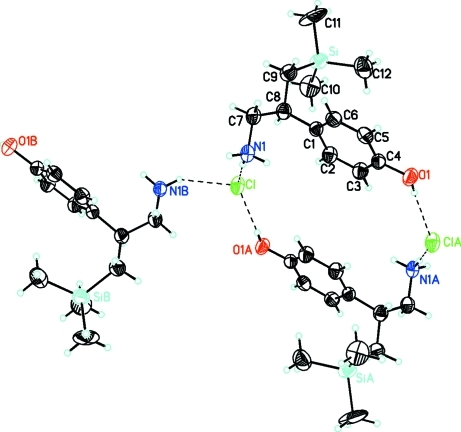
Diagram showing the R24(22) ring motif as well as an additional NH3+···Cl- hydrogen bond [molecule A generated by symmetry code; 2 - x, -y, 1 - z, and molecule B by; 3/2 - x, y - 1/2, 3/2 - z]. Hydrogen bonds are shown by dashed lines.

**Fig. 3.**
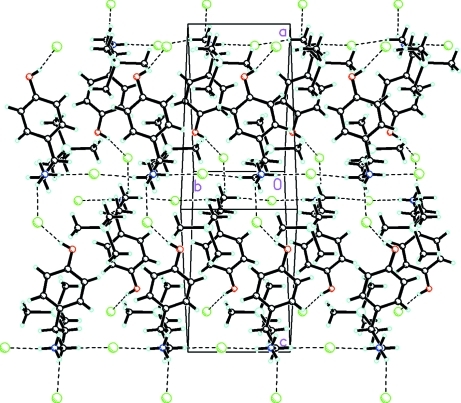
The molecular packing showing the 2-D network of ions linked by O—H···Cl- and N—H···.Cl- hydrogen bonds (shown by dashed lines).

## supplementary crystallographic information

### Comment

The title compound is a substituted α-phenyethylaminium chloride. Phenylethyl amines are substrates for dopamine-β-hydroxylase and are of biological importance. Silicon substituted phenylethyl amines have been investigated for biological activity and use as insecticides and have applications as pharmaceuticals (Frankel *et al.* 1968). These compounds can be viewed as substituted 3-silylpropylamines, where they have application in monolayer construction and nanotechnology (Li *et al.* 2009) and use in oil recovery *via* reverse ionic liquids (Blasucci *et al.*, 2010). Phenylethyl amines are important building blocks in isoquinoline alkaloid synthesis *via* Pictet–Spengler (Lorenz *et al.* 2010). A related structure has been reported (Hijji *et al.*, 2011).

In view of the importance of these compounds the structure of 4-(2-ammonium-1-trimethylsilanylmethyl-ethyl)-phenol chloride, is reported herein. The title compound is a hydrochloride salt and the Cl^-^ anion forms hydrogen bonds with both the NH_3_^+^ and phenol groups forming R^2^_4_(22) ring motifs (Bernstein *et al.*, 1995) as shown in Fig. 2. In the crystal, anions and cations are linked *via* O—H···Cl, N—H···Cl and N—H···O hydrogen bonds to form a two-dimensional network parallel to (101) as is shown in Fig. 3. The bond lengths (Allen *et al.*, 1987) and angles are in normal ranges.

### Experimental

To a solution of 4-hydroxyphenylacetonitrile (3.0 g, 22.55 mole) dissolved in 20 ml dry THF, cooled in an ice bath, was added (14.5 ml, 23.2 mmol) n-BuLi (1.6 *M*, hexane) drop wise. After the addition was complete the mixture was stirred for 15 minutes then (4.5 g, 26.3 mmol) benzyl bromide was added slowly. 20 ml of THF and 15 ml of HMPA were added to the mixture while the flask was in the ice bath. The mixture was stirred for an additional 1 h in the ice bath and 4 h at RT. Aqueous work up gave a solid, m.p 335–336 K,(3.5 g 70% yield)) of 4-benzyloxyphenylacetonitrile. Alkylation of (2.0 g, 8.97 mmol) (III) by treatment with (6 ml, 9.6 mmol) of n-BuLi (1.6*M*, hexanes) then chloromethyltrimethylsilane (1.14 g, 9.33 mmol) for 2 h at RT and work up to give (1.35 g, 48.7% yield) of 2-(4-benzyloxy-phenyl)-3-trimethylsilyl-propionitrile m.p. 376–377 K. Reduction of (1.0 g, 3.23 mmol) of IV in 10 ml of dry THF with (0.5 ml, 5.0 mmol) of BH3.DMS (10 *M* in DMS) followed by acid hydrolysis with HCl and neutralization with NaOH pellets then product isolation and acidification (HCl) gave a white solid (0.81 g, 72% yield). m.p. 468–469 K of 1-(4-Benzyloxy-phenyl)-2-trimethylsilanyl-ethyl-ammonium chloride. Catalytic hydrogenation of (0.5 g, 1.43 mmol) of in 60 ml of ethanol and 0.2 g Pd/C (10%) gave a white solid (0.25 g, 67% yield) of the title compound. A sample was taken and dissolved in water then the solvent was allowed to evaporate slowly to provide clear crystals of the title compound used for X-ray measurements.

^1^H NMR (DMSO-d_6_, 400 MHz): δ (p.p.m.) = 9.35 (s, 1H),7.50 (br s, 3H) 7.05 (d, 2H, J = 8.48 Hz), 6.72 (d, 2H, J = 8.48 Hz), 2.84 (m, 3H), 0.92 (dd,1 H, J = 14.5, 3.5 Hz), 0.914 (dd, 1 H, J = 14.5, 11.0 Hz), -0.26 (s, 9H) Mass spec: 207 (M—NH_3_Cl), 172, 165, 149, 134, 91, 73 ^13^C NMR (DMSO-d_6_, 100 MHz): δ (p.p.m.) = 156.55, 132.53, 129.22, 115.97, 47.80, 39.39, 21.50, -.836.

### Refinement

H atoms were placed in geometrically idealized positions and constrained to ride on their parent atoms with a C—H distances of 0.93 to 0.97 Å, and O—H distance of 0.82 Å, and N—H distances of 0.89 Å and *U*_iso_(H) = 1.2*U*_eq_(C, N, O) [*U*_iso_(H) = 1.5*U*_eq_(CH_3_)].

### Crystal data

**Table:**

C_12_H_22_NOSi^+^·Cl^−^	*F*(000) = 560
*M**_r_* = 259.85	*D*_x_ = 1.117 Mg m^−^^3^
Monoclinic, *P*2_1_/*n*	Cu *K*α radiation, λ = 1.54184 Å
Hall symbol: -P 2yn	Cell parameters from 1894 reflections
*a* = 14.2611 (4) Å	θ = 5.5–75.6°
*b* = 6.7587 (2) Å	µ = 2.79 mm^−^^1^
*c* = 16.0316 (9) Å	*T* = 295 K
β = 91.252 (3)°	Needle, colorless
*V* = 1544.86 (11) Å^3^	0.44 × 0.18 × 0.06 mm
*Z* = 4	

### Data collection

**Table:**

Oxford Diffraction Xcalibur Ruby Gemini diffractometer	3065 independent reflections
Radiation source: Enhance (Cu) X-ray Source	2023 reflections with *I* > 2σ(*I*)
graphite	*R*_int_ = 0.034
Detector resolution: 10.5081 pixels mm^-1^	θ_max_ = 75.8°, θ_min_ = 5.5°
ω scans	*h* = −17→15
Absorption correction: multi-scan (*CrysAlis PRO*; Oxford Diffraction, 2009)	*k* = −8→5
*T*_min_ = 0.713, *T*_max_ = 1.000	*l* = −17→20
5741 measured reflections	

### Refinement

**Table:**

Refinement on *F*^2^	Primary atom site location: structure-invariant direct methods
Least-squares matrix: full	Secondary atom site location: difference Fourier map
*R*[*F*^2^ > 2σ(*F*^2^)] = 0.061	Hydrogen site location: inferred from neighbouring sites
*wR*(*F*^2^) = 0.244	H-atom parameters constrained
*S* = 1.14	*w* = 1/[σ^2^(*F*_o_^2^) + (0.1021*P*)^2^ + 1.2793*P*] where *P* = (*F*_o_^2^ + 2*F*_c_^2^)/3
3065 reflections	(Δ/σ)_max_ = 0.002
146 parameters	Δρ_max_ = 0.42 e Å^−^^3^
0 restraints	Δρ_min_ = −0.46 e Å^−^^3^

### Special details

**Table:**

Geometry. All e.s.d.'s (except the e.s.d. in the dihedral angle between two l.s. planes) are estimated using the full covariance matrix. The cell e.s.d.'s are taken into account individually in the estimation of e.s.d.'s in distances, angles and torsion angles; correlations between e.s.d.'s in cell parameters are only used when they are defined by crystal symmetry. An approximate (isotropic) treatment of cell e.s.d.'s is used for estimating e.s.d.'s involving l.s. planes.
Refinement. Refinement of *F*^2^ against ALL reflections. The weighted *R*-factor *wR* and goodness of fit *S* are based on *F*^2^, conventional *R*-factors *R* are based on *F*, with *F* set to zero for negative *F*^2^. The threshold expression of *F*^2^ > σ(*F*^2^) is used only for calculating *R*-factors(gt) *etc*. and is not relevant to the choice of reflections for refinement. *R*-factors based on *F*^2^ are statistically about twice as large as those based on *F*, and *R*- factors based on ALL data will be even larger.

### Fractional atomic coordinates and isotropic or equivalent isotropic displacement parameters (Å^2^)

**Table:**

	*x*	*y*	*z*	*U*_iso_*/*U*_eq_	
Cl	0.79604 (9)	−0.16189 (18)	0.66227 (8)	0.0740 (4)	
Si	0.60028 (10)	0.1988 (3)	0.36494 (9)	0.0772 (5)	
O1	1.0447 (2)	0.4507 (5)	0.3547 (2)	0.0716 (9)	
H1	1.0773	0.3522	0.3484	0.107*	
N1	0.7889 (3)	0.2955 (6)	0.6591 (2)	0.0607 (9)	
H1A	0.7848	0.3526	0.7090	0.091*	
H1B	0.8399	0.3391	0.6340	0.091*	
H1C	0.7926	0.1649	0.6654	0.091*	
C1	0.8001 (3)	0.3071 (6)	0.4778 (2)	0.0532 (9)	
C2	0.8626 (3)	0.1619 (7)	0.4569 (3)	0.0631 (11)	
H2A	0.8498	0.0312	0.4706	0.076*	
C3	0.9446 (3)	0.2058 (7)	0.4155 (3)	0.0672 (12)	
H3A	0.9856	0.1049	0.4011	0.081*	
C4	0.9650 (3)	0.3994 (7)	0.3960 (3)	0.0566 (10)	
C5	0.9045 (3)	0.5478 (7)	0.4181 (3)	0.0611 (10)	
H5A	0.9185	0.6789	0.4060	0.073*	
C6	0.8227 (3)	0.5012 (7)	0.4584 (3)	0.0627 (11)	
H6A	0.7819	0.6023	0.4728	0.075*	
C7	0.7046 (3)	0.3453 (7)	0.6075 (3)	0.0654 (11)	
H7A	0.6992	0.4880	0.6029	0.078*	
H7B	0.6491	0.2965	0.6348	0.078*	
C8	0.7093 (3)	0.2553 (7)	0.5201 (3)	0.0636 (11)	
H8A	0.7077	0.1111	0.5264	0.076*	
C9	0.6207 (4)	0.3158 (8)	0.4699 (3)	0.0745 (13)	
H9A	0.6225	0.4580	0.4622	0.089*	
H9B	0.5668	0.2868	0.5037	0.089*	
C10	0.6136 (6)	−0.0743 (10)	0.3729 (5)	0.119 (2)	
H10A	0.6038	−0.1327	0.3188	0.179*	
H10B	0.5683	−0.1257	0.4106	0.179*	
H10C	0.6756	−0.1055	0.3934	0.179*	
C11	0.4808 (4)	0.2622 (16)	0.3295 (6)	0.156 (4)	
H11A	0.4686	0.2045	0.2756	0.234*	
H11B	0.4748	0.4034	0.3257	0.234*	
H11C	0.4367	0.2119	0.3685	0.234*	
C12	0.6839 (5)	0.2972 (12)	0.2874 (4)	0.108 (2)	
H12A	0.6725	0.2343	0.2344	0.163*	
H12B	0.7471	0.2704	0.3061	0.163*	
H12C	0.6753	0.4374	0.2816	0.163*	

### Atomic displacement parameters (Å^2^)

**Table:**

	*U*^11^	*U*^22^	*U*^33^	*U*^12^	*U*^13^	*U*^23^
Cl	0.0907 (9)	0.0624 (7)	0.0700 (7)	0.0036 (6)	0.0276 (6)	−0.0015 (5)
Si	0.0625 (8)	0.0911 (11)	0.0776 (9)	0.0034 (7)	−0.0070 (6)	−0.0219 (8)
O1	0.0607 (18)	0.075 (2)	0.080 (2)	−0.0050 (16)	0.0195 (15)	0.0089 (18)
N1	0.067 (2)	0.062 (2)	0.0531 (18)	−0.0084 (18)	0.0123 (16)	−0.0044 (16)
C1	0.053 (2)	0.061 (2)	0.0455 (19)	−0.0004 (18)	0.0028 (15)	−0.0042 (17)
C2	0.071 (3)	0.053 (2)	0.066 (3)	−0.004 (2)	0.010 (2)	0.001 (2)
C3	0.072 (3)	0.058 (2)	0.072 (3)	0.004 (2)	0.013 (2)	0.001 (2)
C4	0.054 (2)	0.066 (2)	0.050 (2)	−0.0039 (19)	0.0031 (16)	0.0018 (18)
C5	0.073 (3)	0.054 (2)	0.056 (2)	−0.002 (2)	−0.0016 (19)	0.0050 (19)
C6	0.069 (3)	0.060 (2)	0.059 (2)	0.010 (2)	0.0017 (19)	−0.002 (2)
C7	0.073 (3)	0.068 (3)	0.056 (2)	0.001 (2)	0.009 (2)	−0.006 (2)
C8	0.068 (3)	0.066 (3)	0.057 (2)	−0.005 (2)	0.0051 (19)	−0.006 (2)
C9	0.071 (3)	0.082 (3)	0.070 (3)	0.002 (3)	0.001 (2)	−0.012 (3)
C10	0.139 (6)	0.090 (5)	0.130 (6)	−0.005 (5)	0.014 (5)	−0.019 (4)
C11	0.042 (3)	0.243 (11)	0.182 (8)	0.042 (4)	−0.031 (4)	−0.097 (8)
C12	0.127 (6)	0.128 (6)	0.070 (3)	−0.010 (5)	−0.005 (3)	−0.007 (4)

### Geometric parameters (Å, °)

**Table:**

Si—C11	1.835 (6)	C5—H5A	0.9300
Si—C10	1.860 (7)	C6—H6A	0.9300
Si—C12	1.865 (7)	C7—C8	1.532 (6)
Si—C9	1.876 (5)	C7—H7A	0.9700
O1—C4	1.372 (5)	C7—H7B	0.9700
O1—H1	0.8200	C8—C9	1.537 (7)
N1—C7	1.484 (6)	C8—H8A	0.9800
N1—H1A	0.8900	C9—H9A	0.9700
N1—H1B	0.8900	C9—H9B	0.9700
N1—H1C	0.8900	C10—H10A	0.9600
C1—C2	1.373 (6)	C10—H10B	0.9600
C1—C6	1.389 (6)	C10—H10C	0.9600
C1—C8	1.516 (6)	C11—H11A	0.9600
C2—C3	1.388 (6)	C11—H11B	0.9600
C2—H2A	0.9300	C11—H11C	0.9600
C3—C4	1.379 (6)	C12—H12A	0.9600
C3—H3A	0.9300	C12—H12B	0.9600
C4—C5	1.375 (6)	C12—H12C	0.9600
C5—C6	1.381 (6)		
			
C11—Si—C10	110.2 (4)	N1—C7—H7B	109.3
C11—Si—C12	108.3 (4)	C8—C7—H7B	109.3
C10—Si—C12	109.5 (4)	H7A—C7—H7B	107.9
C11—Si—C9	107.7 (3)	C1—C8—C7	111.8 (4)
C10—Si—C9	110.1 (3)	C1—C8—C9	113.9 (4)
C12—Si—C9	111.1 (3)	C7—C8—C9	108.7 (4)
C4—O1—H1	109.5	C1—C8—H8A	107.3
C7—N1—H1A	109.5	C7—C8—H8A	107.3
C7—N1—H1B	109.5	C9—C8—H8A	107.3
H1A—N1—H1B	109.5	C8—C9—Si	117.8 (3)
C7—N1—H1C	109.5	C8—C9—H9A	107.9
H1A—N1—H1C	109.5	Si—C9—H9A	107.9
H1B—N1—H1C	109.5	C8—C9—H9B	107.9
C2—C1—C6	117.7 (4)	Si—C9—H9B	107.9
C2—C1—C8	120.7 (4)	H9A—C9—H9B	107.2
C6—C1—C8	121.6 (4)	Si—C10—H10A	109.5
C1—C2—C3	121.5 (4)	Si—C10—H10B	109.5
C1—C2—H2A	119.2	H10A—C10—H10B	109.5
C3—C2—H2A	119.2	Si—C10—H10C	109.5
C4—C3—C2	119.7 (4)	H10A—C10—H10C	109.5
C4—C3—H3A	120.2	H10B—C10—H10C	109.5
C2—C3—H3A	120.2	Si—C11—H11A	109.5
O1—C4—C5	118.1 (4)	Si—C11—H11B	109.5
O1—C4—C3	122.0 (4)	H11A—C11—H11B	109.5
C5—C4—C3	119.8 (4)	Si—C11—H11C	109.5
C4—C5—C6	119.7 (4)	H11A—C11—H11C	109.5
C4—C5—H5A	120.2	H11B—C11—H11C	109.5
C6—C5—H5A	120.2	Si—C12—H12A	109.5
C5—C6—C1	121.5 (4)	Si—C12—H12B	109.5
C5—C6—H6A	119.2	H12A—C12—H12B	109.5
C1—C6—H6A	119.2	Si—C12—H12C	109.5
N1—C7—C8	111.7 (4)	H12A—C12—H12C	109.5
N1—C7—H7A	109.3	H12B—C12—H12C	109.5
C8—C7—H7A	109.3		
			
C6—C1—C2—C3	−1.7 (7)	C6—C1—C8—C7	−63.8 (5)
C8—C1—C2—C3	177.7 (4)	C2—C1—C8—C9	−119.4 (5)
C1—C2—C3—C4	1.0 (7)	C6—C1—C8—C9	60.0 (6)
C2—C3—C4—O1	−179.6 (4)	N1—C7—C8—C1	−52.4 (5)
C2—C3—C4—C5	0.5 (7)	N1—C7—C8—C9	−179.1 (4)
O1—C4—C5—C6	178.8 (4)	C1—C8—C9—Si	62.4 (5)
C3—C4—C5—C6	−1.3 (7)	C7—C8—C9—Si	−172.2 (4)
C4—C5—C6—C1	0.5 (7)	C11—Si—C9—C8	169.6 (5)
C2—C1—C6—C5	1.0 (7)	C10—Si—C9—C8	49.4 (5)
C8—C1—C6—C5	−178.4 (4)	C12—Si—C9—C8	−72.0 (5)
C2—C1—C8—C7	116.8 (5)		

### Hydrogen-bond geometry (Å, °)

**Table:**

*D*—H···*A*	*D*—H	H···*A*	*D*···*A*	*D*—H···*A*
O1—H1···Cl^i^	0.82	2.23	3.012 (4)	160.
N1—H1A···Cl^ii^	0.89	2.39	3.146 (3)	143.
N1—H1B···O1^iii^	0.89	2.18	2.941 (5)	143.
N1—H1C···Cl	0.89	2.21	3.093 (4)	172.

Symmetry codes: (i) −*x*+2, −*y*, −*z*+1; (ii) −*x*+3/2, *y*+1/2, −*z*+3/2; (iii) −*x*+2, −*y*+1, −*z*+1.
